# Impact of Adlay-Based Formula on Pain and Discomfort in Women with Dysmenorrhea: A Randomized Controlled Trial

**DOI:** 10.3390/nu16234026

**Published:** 2024-11-24

**Authors:** Yi-Fen Chiang, Ko-Chieh Huang, Mohamed Ali, Shih-Min Hsia

**Affiliations:** 1 School of Nutrition and Health Sciences, College of Nutrition, Taipei Medical University, Taipei 11031, Taiwan; da07108002@tmu.edu.tw (Y.-F.C.); da07111001@tmu.edu.tw (K.-C.H.); 2 Clinical Pharmacy Department, Faculty of Pharmacy, Ain Shams University, Cairo 11566, Egypt; mohamed.ali@bsd.uchicago.edu; 3 Department of Obstetrics and Gynecology, University of Chicago, Chicago, IL 60637, USA; 4 Graduate Institute of Metabolism and Obesity Sciences, College of Nutrition, Taipei Medical University, Taipei 11031, Taiwan; 5 School of Food Safety, Taipei Medical University, Taipei 11031, Taiwan; 6 Nutrition Research Center, Taipei Medical University Hospital, Taipei 11031, Taiwan; 7 TMU Research Center for Digestive Medicine, Taipei Medical University, Taipei 110301, Taiwan

**Keywords:** adlay-based formula, primary dysmenorrhea, inflammatory biomarkers, painfulness

## Abstract

Background: Primary dysmenorrhea, a highly prevalent condition that significantly impacts women’s daily activities and quality of life, occurs without underlying pelvic pathological changes. Conventional treatments, such as warm water therapy, provide temporary relief; however, more effective interventions are needed. This study aimed to evaluate the effectiveness of an Adlay-based formula in reducing dysmenorrhea symptoms through randomized controlled trials. Methods: A total of 69 participants were randomly assigned to either the Adlay-based formula group (*n* = 35) or the placebo group (*n* = 34). Baseline characteristics, including age, age of menarche, dysmenorrhea onset, menstrual duration, BMI, blood pressure, and heart rate, were comparable between groups. The primary outcomes were measured using the Visual Analogue Scale (VAS) for dysmenorrhea, pain assessment scales, the Menstrual Distress Questionnaire (MDQ), and serum levels of inflammatory biomarkers (PGE2, PGF2α, IL-6, Hs-CRP). Results: The intervention group showed a significant reduction in VAS scores at both the first treatment and at the end of the study compared to baseline and the placebo group. Pain assessments indicated improvements in persistent pain, dull pain, exhaustion, nausea/vomiting, lower abdominal swelling, back pain, diarrhea, and cold sweats. Additionally, biomarker analysis revealed significant reductions in PGE2, PGF2α, and Hs-CRP levels in the intervention group, with no significant change in IL-6 levels. Conclusions: The Adlay-based formula effectively alleviated dysmenorrhea symptoms, improved pain and discomfort, and reduced inflammatory biomarkers compared to placebo. These findings suggested that the formula could serve as a promising alternative for managing primary dysmenorrhea.

## 1. Introduction

Dysmenorrhea is the most prevalent gynecological disorder among reproductive-aged women worldwide, with a prevalence of over 90% [1,2,3]. This disease often results in absenteeism from school or work, negatively impacting performance, depression, and causing economic losses, which all collectively result in decreased quality of life [4].

Dysmenorrhea is classified into primary and secondary types. Primary dysmenorrhea refers to menstrual pain without any underlying pelvic pathology. In contrast, secondary dysmenorrhea is associated with pelvic pathologies such as endometriosis, adenomyosis, or leiomyoma which are identified through abnormal pelvic examinations using ultrasound or Magnetic Resonance Imaging (MRI) [5,6]. The painful symptoms of primary dysmenorrhea often begin in a woman’s late teens or early twenties and are particularly prevalent among women of reproductive age [7]. Dysmenorrhea is characterized by lower abdominal pain, often accompanied by symptoms such as headache, diarrhea, and vomiting. The pain and discomfort typically begin with the onset of menstrual flow and can persist for 8 to 72 h [4]. The exact cause of dysmenorrhea remains unclear, though it is widely believed to be related to excessive uterine contractions with elevating levels of prostaglandin (PG) F2α during menstruation [8,9]. PGs are produced by cyclooxygenase-2 (COX-2) and can induce uterine vasoconstriction, leading to ischemia. Additionally, PGs can sensitize afferent nerves, which contribute to dysmenorrhea-associated pain [9,10]. Finally, it was suggested that these PGs enhance uterine contractility and induce cramping pain through temporary increases in uterine pressure [11].

Conventional treatment for primary dysmenorrhea often involves the use of non-steroidal anti-inflammatory drugs (NSAIDs) for three menstrual cycles. These medications work by inhibiting prostaglandin production, thereby alleviating pain and associated symptoms [11,12]. NSAIDs, such as ibuprofen, aspirin, and naproxen, provide anti-inflammatory, analgesic, and antipyretic effects by inhibiting COX-2 formation [13]. However, they also have several side effects, including headaches, dizziness, nausea, and indigestion, and with long-term use, it can lead to drug resistance [14]. For individuals, who do not respond to NSAID treatment, oral contraceptive pills containing estrogen or progestin are often prescribed for three menstrual cycles, as a safe and effective option for managing adolescent dysmenorrhea [7]. These contraceptives modulate endometrial tissue growth and reduce PG levels, thereby alleviating inflammation and improving pain symptoms [15]. In addition to pharmacological approaches, alternative interventions such as herbal remedies [16], exercise [17], heat therapy [18], aromatherapy [19], and dietary supplements [2,20] have demonstrated potential benefits in alleviating the symptoms of dysmenorrhea.

Adlay (*Coix lachryma-jobi* L. var. *ma-yuen* Stapf.), commonly known as Job’s tears, is a traditional Chinese medicinal plant and food supplement. It has demonstrated beneficial effects on reproductive health, as well as possessing anti-cancer and antioxidant properties [21,22,23,24]. Our previous study highlighted the alleviating effects of adlay extract on uterine smooth muscle contractions and elucidated the underlying PGF_2α_-induced uterine contractions and intracellular Ca^2+^ mobilization in both in vitro and in vivo study [25]. To further explore the therapeutic potential of adlay, we have developed an adlay extract-based formula. This formula will be evaluated for its efficacy in alleviating dysmenorrhea symptoms in the current study.

## 2. Material and Methods

### 2.1. Study Design and Enrollment

Participants were recruited through university-wide poster campaigns and social media platforms targeted at females experiencing dysmenorrhea. Upon expressing interest, participants were screened via a preliminary questionnaire to confirm eligibility based on the inclusion and exclusion criteria.

The inclusion criteria specified females aged 20–40 years, who reported a recent history of painful menstruation, defined as experiencing moderate to severe pain that disrupted daily activities during the first or second day of menstruation within the past six months. All participants had regular menstrual cycles of 21–35 days, with menstruation lasting 3–7 days.

The exclusion criteria included pregnancy, breastfeeding, plans to conceive, current use of contraceptives, recent use of painkillers or anti-inflammatory drugs, a history of uterine or ovarian surgery, severe pelvic inflammatory disease (such as endometriosis or uterine leiomyoma), and major illnesses, including cancer, heart disease, kidney disease, thyroid dysfunction, or hormone treatments for menstrual pain within the past month.

Participants, who met the criteria, underwent blood sampling (10 mL) during their menstrual cycle. The collected samples were centrifuged at 4000 rpm for 10 min at 4 °C, after which serum was separated and stored at −80 °C until analysis.

The trial was approved by the TMU-Joint Institutional Review Board (TMU-JIRB, N201708023) and was registered at ClinicalTrials.gov (NCT number: NCT06637553). All participants provided written informed consent prior to the commencement of the study.

During the experimental period, participants consumed 20 g of hot water extract of adlay daily for two menstrual cycles for 7 days. The adlay extract-based formula, composed of grape seed, cinnamon, and onion extract, is recognized as a safe food material with no known side effects. The participants were monitored for any adverse reactions, and no side effects were reported during the intervention.

### 2.2. Formula Preparation

The adlay used in the formula was sourced from the Taichung District Agricultural Research and Extension Station in Taichung, Taiwan, and ground into a 20-mesh powder. The 70% ethanol extract was prepared following a previous study [26]. The formula also includes onion, grape seed, and cinnamon extracts, with all components provided by A.T.P. CO., LTD., Taipei, Taiwan.

### 2.3. Randomization and Blinding

To ensure proper blinding, the placebo was made to resemble the treatment supplement in appearance, taste, and packaging. Participants were not informed of the treatment group assignments, and efforts were made to prevent them from discerning whether they were receiving the placebo or the actual supplement.

The adlay extract formula and placebo powders were placed in similar unlabeled bags, labeled as “code A” or “code B”. A programmer generated a randomization list using Microsoft Excel software. Researchers, participants, and statistical analysts were blinded to the group assignments until the results were analyzed.

### 2.4. Cytokines Evaluation

Serum levels of PGE2, PGF_2α_, and IL-6 were examined using an ELISA kit (Cayman, Ann Arbor, MI, USA), following the manufacturer’s procedure.

### 2.5. Painfulness and Discomfort Scale Evaluation

The evaluation of dysmenorrheal pain and menstrual discomfort was conducted using two structured questionnaires [27]. Moreover, we used a Visual Analogue Scale (VAS), ranging from 0 to 10, to evaluate the painfulness improvement.

The pain assessment scale assessed the location and severity of menstrual pain across 15 different pain types, including bursts of throbbing pain, sudden severe pain, needle-like pain, cutting pain, spasm and colic, pain like being gnawed away, burning pain, persistent pain, dull pain, pain when touched, tearing pain, feeling of exhaustion, nausea and vomiting, fear and dread, and suffering from cruel pain. Pain severity was categorized as none, mild, moderate, or severe.

The Menstrual Distress Questionnaire (MDQ) measured the degree of discomfort experienced during menstruation across 15 different symptoms, including dizziness, muscle soreness, breast pain or swelling, swelling in the lower abdomen, general aches and pains, back pain, headache, skin problems, nausea or vomiting, diarrhea, burnout, weight gain, cheeks that are hot and red, cold sweat, and heart palpitations. Discomfort severity was rated on a scale from 1 (no symptoms) to 4 (severe discomfort). Both tools were validated for reliability and they provided a comprehensive assessment of the participants’ experiences during their menstrual cycles. The raw data for the score evaluations can be found in Supplementary Tables S1 and S2.

### 2.6. Statistical Analysis

Data from the study were statistically analyzed using GraphPad Prism (version 8.0). Statistical significance was determined using student’s *t* tests, and results were presented as mean ± standard deviation. A *p*-value of less than 0.05 was considered statistically significant.

## 3. Results

**Figure 1:**
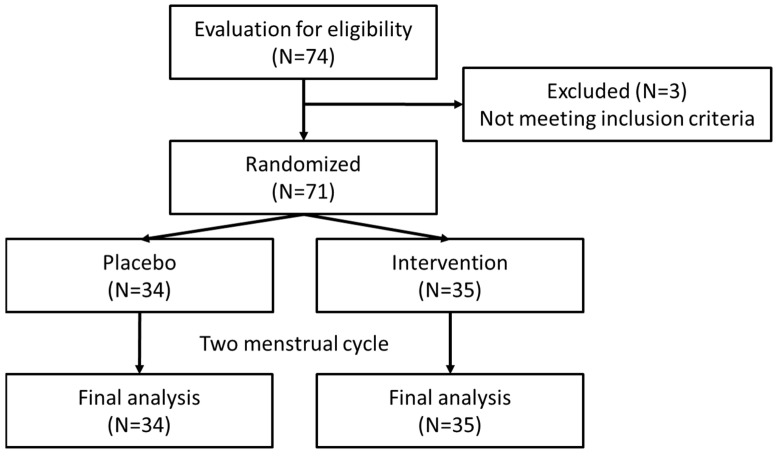
Flowchart of trial.

After enrollment, the baseline characteristics of the study population were compared between the intervention group (N = 35) and the placebo group (N = 34) for further analysis (Figure 1). The mean age of participants, mean age of menarche, mean age at which participants experienced dysmenorrhea, duration of the menstrual period, and mean BMI were similar between the two groups with no statistically significant differences as well as differences in systolic blood pressure (SBP) and diastolic blood pressure (DBP) between the groups. However, the heart rate (HR) was significantly higher in the intervention group (82.9 ± 12.21 bpm) compared to the placebo group (75.3 ± 10.08 bpm), with a *p* value of 0.006 (Table 1).

**Figure 2:**
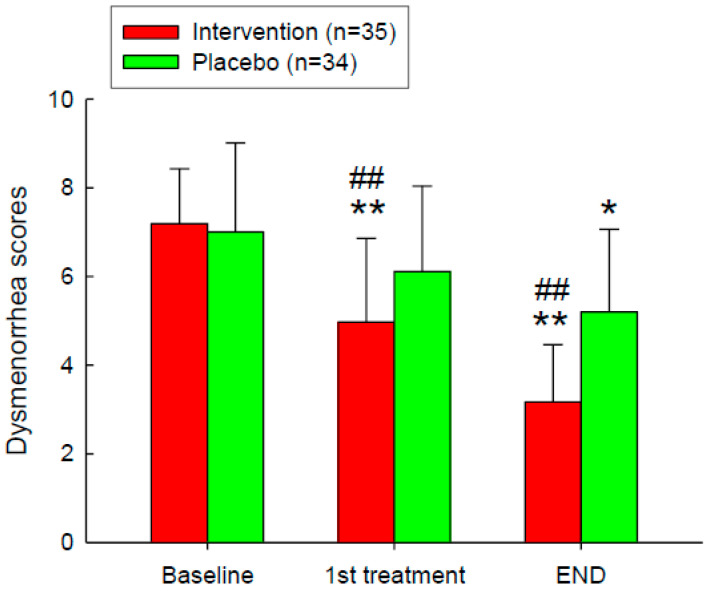
VAS Painfulness Improvement After the Intervention. Intervention on dysmenorrhea scores, measured using the Visual Analogue Scale (VAS), for the intervention group (*n* = 35, red bars) and the placebo group (*n* = 34, green bars) at three-time points: Baseline, 1st Treatment, and End. *, *p* < 0.05; **, *p* < 0.01 compared with the baseline. ##, *p* < 0.01 compared with the placebo group. Error bars represent standard deviations.

The effect of Adlay intervention on VAS scores among participants, in both intervention groups (*n* = 35) with a placebo group (*n* = 34) was evaluated (Figure 2). Scores, ranging from 0 to 10, were evaluated at three-time points: Baseline, 1st Treatment, and End. The intervention group is represented by red bars, while the placebo group is represented by green bars. At baseline, both groups had similar VAS scores. After the first treatment, the intervention group exhibited a significant reduction in VAS scores compared to both the baseline and the placebo group. By the end of the study, the intervention group maintained significantly lower dysmenorrhea scores compared to both their baseline scores and the placebo group.

**Figure 3:**
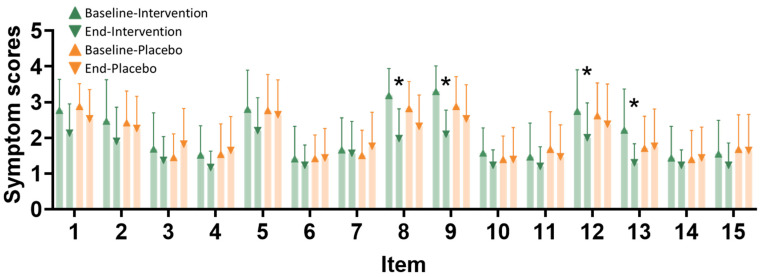
Painfulness item improvement after the intervention. Pain assessment scales were evaluated at baseline and at the endpoint after the intervention. Item types are as follows: 1—bursts of throbbing pain, 2—sudden severe pain, 3—needle-like pain, 4—cutting pain, 5—spasm and colic, 6—pain like being gnawed away, 7—burning pain, 8—persistent pain, 9—dull pain, 10—pain when touched, 11—tearing pain, 12—feeling of exhaustion, 13—nausea and vomiting, 14—fear and dread, and 15—suffering from cruel pain. The *x*-axis represents different symptoms (1–15), and the *y*-axis shows the symptom scores. The intervention group (*n* = 35) is represented by green triangles at baseline and green inverted triangles at the end of the study. The placebo group (*n* = 34) is represented by orange triangles at baseline and orange inverted triangles at the end of the study. *, *p* < 0.05 compared with the baseline after the intervention.

The pain assessment scales were evaluated at baseline and at the endpoint after the intervention. Symptom scores range from 1 to 4, with higher scores indicating greater severity of symptoms (Figure 3). Significant changes in symptom scores between baseline and the end of the study, within and between the groups, are observed for the following symptoms: persistent pain, dull pain, exhaustion, and vomiting/nausea.

**Figure 4:**
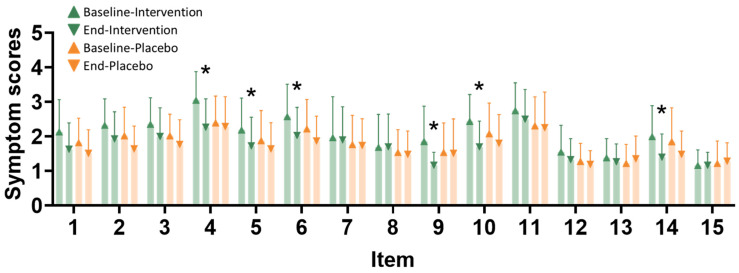
Discomfort item improvement after the intervention. Discomfort assessment scales were evaluated at baseline and at the endpoint after the intervention. Item types are as follows: 1—dizziness, 2—muscle soreness, 3—breast pain or swelling, 4—swelling in the lower abdomen, 5—general aches and pains, 6—back pain, 7—headache, 8—skin problems, 9—nausea or vomiting, 10—diarrhea, 11—burnout, 12—weight gain, 13—cheeks that are hot and red, 14—cold sweat, and 15—heart palpitations. The icons are the same as in the VAS evaluation. The *x*-axis represents different symptoms (1–15). The intervention group (*n* = 35) is represented by green triangles at baseline and green inverted triangles at the end of the study. The placebo group (*n* = 34) is represented by orange triangles at baseline and orange inverted triangles at the end of the study. * indicates *p* < 0.05 compared with the baseline after the intervention.

The Menstrual Distress Questionnaire (MDQ) was used to compare between intervention and placebo groups at baseline and end of study (Figure 4). Significant changes in symptom scores between baseline and the end of the study, within and between the groups, are observed for the following symptoms: swelling in the lower abdomen, aches and pains, back pain, nausea, diarrhea, and cold sweat.

Next, we evaluated the effects of Adlay intervention on the serum levels of various biomarkers in the placebo group (*n* = 34) as compared to the intervention group (*n* = 35) (Table 2). The baseline comparison showed no statistically significant differences between the two groups in the levels of PGE2, PGF_2α_, IL-6, or Hs-CRP. After the first treatment, significant reductions were observed in PGE2 (1292.8 ± 764.1 vs. 2177.8 ± 770.8 pg/mL, *p* < 0.001) and Hs-CRP (1542.8 ± 561.6 vs. 2232.5 ± 663.6 ng/mL, *p* = 0.009) in the intervention group compared to the placebo group. By the end of the study, the intervention group showed significant reductions in PGE2 (1126.4 ± 1088.2 vs. 1623.4 ± 619.6 pg/mL, *p* = 0.009), PGF_2α_ (1116.7 ± 771.7 vs. 1912.3 ± 902.6 pg/mL, *p* = 0.008), and Hs-CRP (1112.1 ± 253.2 vs. 2321.1 ± 353.2 ng/mL, *p* < 0.001). IL-6 levels did not show significant changes at any measured time point. These results suggest that Adlay intervention significantly reduced inflammatory biomarkers, particularly PGE2, PGF_2α_, and Hs-CRP, compared to the placebo, and the reduction was strengthened over time.

## 4. Discussion

This study represents the first trial for the utility of an adlay-based formula in patients with dysmenorrhea. The intervention, conducted over two menstrual cycles, significantly minimized the pain and discomfort associated with dysmenorrhea. Additionally, it effectively reduced the serum levels of dysmenorrhea-related PGE2, PGF2α, and hs-CRP cytokines. Overall, our data demonstrate the beneficial properties of the adlay-based formula on dysmenorrhea and highlight its potential to serve as a viable alternative treatment for dysmenorrhea with the advantages of offering relief without the side effects usually associated with conventional NSAIDs.

Nearly 93% of afflicted women reported experiencing pain with every menstrual period with more than 41% reporting a significant negative impact on their daily activities due to the pain. Emotional fluctuations during this time are often attributed to menstrual pain, which varies in intensity and frequency but generally causes some level of discomfort [28].

In terms of pain management, A survey results showed that the most common method was drinking beverages such as brown sugar water, longan tea, or hot cocoa to reduce the pain. The second most common method, almost equal in prevalence at 78.2%, was resting and not doing anything. Other less commonly used methods were eating chocolate, drinking warm water, or applying heat. Importantly, the preference for utilizing non-pharmaceutical methods was nearly 43.8%. These findings suggest that many women would prefer to alleviate menstrual pain through dietary means rather than medications, highlighting the potential room for exploring non-pharmaceutical remedies [29]. This preference for natural remedies over medication highlights the importance of providing effective and accessible dietary options for menstrual pain relief. Our explored formula, intended to be dissolved in warm water, offers an effective alternative to conventional warm water treatments, providing a convenient and natural method for relieving menstrual pain.

During menstrual bleeding, the demise of the corpus luteum triggers progesterone secretion, which stimulates inflammation and prostaglandin production in the endometrium, with downstream inflammatory response [30]. In the first few days of the menstrual cycle, a significant elevation of PGF2α is observed in patients experiencing menstrual pain [31]. Our previous study demonstrated that adlay extract could counteract PGF2α-induced uterine contractions. By modulating calcium influx, the extract effectively reduced the signaling transduction involved in uterine contractions. Furthermore, the addition of onion, cinnamon, and grape seed extracts enhanced the formula’s effectiveness. The active compounds, quercetin, and resveratrol, exhibited significant effects through calcium modulation and showed potential inhibition of contractions induced by various agonists such as PGF2α, oxytocin, carbachol, and high K+ solutions [25,32].

Moreover, the Adlay hull extract has demonstrated significant anti-inflammatory potential, attributed to its abundance of bioactive compounds, including eriodictyol, the ceramide (2S,3S,4R)-2-[(2′R)-2′-hydroxytetracosanoyl-amino]-1,3,4-octadecanetriol, and p-coumaric acid. These compounds effectively inhibited LPS-induced production of nitric oxide (NO) and PGE2 in RAW 264.7 macrophages, highlighting the Adlay extract’s potential as a key modulator of inflammation [33].

The intervention of dietary supplements in managing dysmenorrhea works through the modulation of nutritional and biochemical pathways to alleviate pain and discomfort. Supplements, including vitamins [34,35], minerals [36], herbs [37], and botanical extracts [38,39], are widely used as complementary and alternative medicine (CAM) therapies. Traditionally, these supplements are available in the form of tablets, capsules, soft gels, and gel caps, providing convenient self-administration [40]. However, while these forms offer relief, they may not fully address the practical needs of those seeking immediate comfort during painful menstrual periods.

To enhance both efficacy and convenience, we have developed a hot drink-based formula specifically designed for dysmenorrhea relief. This approach not only provides the therapeutic benefits of dietary supplements but also offers soothing warmth, which is known to alleviate menstrual pain [41]. The hot drink format delivers an innovative, practical solution, making it easier to integrate supplement intake into daily routines, especially during periods of discomfort. This formulation aims to combine the advantages of CAM therapies with the added comfort and immediate relief of a warm beverage, providing a comprehensive approach to dysmenorrhea management.

This study has several limitations. While blood measurements were used to assess the relationship between dysmenorrhea and prostaglandins, this provides only an indirect correlation. Elevated prostaglandin levels are typically observed within the endometrium of dysmenorrhea patients, and future studies should consider direct endometrial tissue analysis to better capture this relationship.

Another important limitation of this study is the small sample size, which may restrict the generalizability of our findings. Larger and more diverse study populations are necessary for future research to validate our results and further explore the potential of the Adlay-based formula in managing dysmenorrhea. Additionally, the exclusion of secondary dysmenorrhea, particularly endometriosis-related cases, was based on participants’ self-reported medical history rather than objective diagnostic tools such as ultrasound or MRI [6]. This approach may introduce inaccuracies and underscores the need for more rigorous diagnostic methods in subsequent studies. Finally, there remains uncertainty regarding the exact bioactive compounds responsible for the observed effects. Identifying these active components is essential to fully understand the treatment’s mechanism of action and optimize its clinical application. Collectively, we demonstrated that the combined formulation of adlay extract with these additional plant extracts offers a multifaceted approach to managing dysmenorrhea. The inhibition of calcium influx plays a crucial role in reducing uterine contractions and subsequent menstrual pain.

## 5. Conclusions

Our study demonstrates the effectiveness of an Adlay-based formula in alleviating the symptoms of primary dysmenorrhea. By reducing serum levels of PGE2, PGF2α, and hs-CRP, and improving pain-related parameters, the formula addresses key inflammatory and contractile pathways associated with dysmenorrhea. Importantly, this innovative hot drink formulation offers a natural, convenient, and effective alternative that aligns with the preference of many women for dietary interventions over pharmaceutical options.

The dual benefits of warmth and anti-inflammatory action position this formula as a promising candidate for managing menstrual pain. However, further research is necessary to identify the precise bioactive components responsible for its effects and to evaluate its long-term efficacy in larger, more diverse populations. These efforts will not only refine its clinical applications but also advance personalized and non-pharmaceutical strategies for dysmenorrhea management.

## Figures and Tables

**Table 1:** Baseline Characteristics of the Study Population.

Variable	Intervention (N = 35)	Placebo (N = 34)	*p* Value
Age (year)	23.03 ± 3.69	21.57 ± 1.31	0.077
Age of menarche (year)	12.06 ± 1.35	12.14 ± 1.19	0.501
Age of dysmenorrhea (year)	14.03 ± 0.56	13.86 ± 0.81	0.338
Menstrual period	6.28 ± 0.74	6.31 ± 0.63	0.99
BMI	20.78 ± 2.81	20.44 ± 2.47	0.836
Systolic blood pressure (SBP)	111.03 ± 12.15	107.94 ± 11.32	0.273
Diastolic blood pressure (DBP)	70.42 ± 9.57	67.91 ± 11.57	0.324
Heart rate (HR)	82.9 ± 12.21	75.3 ± 10.08	0.006

**Table 2:** Effects of adlay’s products on serum levels of biochemical parameters in patients with primary dysmenorrhea at baseline and after the intervention period.

Variable		Intervention N = 35	Placebo N = 34	*p* Value ^1^	*p* Value ^2^	*p* Value ^3^
PGE2 (pg/mL)	Baseline	1717.1 ± 1167.1	1832.1 ± 692.4	0.129	-	-
	1st treatment	1292.8 ± 764.1	2177.8 ± 770.8	<0.001	0.138	0.077
	End	1126.4 ± 1088.2	1623.4 ± 619.6	0.009	0.019	0.22
PGF_2α_ (pg/mL)	Baseline	2046.3 ± 1329.9	1839.3 ± 980.0	0.826	-	-
	1st treatment	1518.6 ± 1009.3	1692.3 ± 1449.7	0.797	0.1	0.17
	End	1116.7 ± 771.7	1912.3 ± 902.6	0.008	0.032	0.116
IL-6 (pg/mL)	Baseline	27.4 ± 8.6	29.4 ± 10.6	0.781	-	-
	1st treatment	32.5 ± 21.4	31.9 ± 17.7	0.852	0.874	0.653
	End	29.4 ± 11.9	28.5 ± 14.6	0.807	0.912	0.788
Hs-CRP (ng/mL)	Baseline	2756.3 ± 881.7	2565.1 ± 453.2	0.99	-	-
	1st treatment	1542.8 ± 561.6	2232.5 ± 663.6	0.009	0.012	0.569
	End	1112.1 ± 253.2	2321.1 ± 353.2	<0.001	0.004	0.815

*p* value ^1^: between-group comparison of variables resulted from independent sample *t*-test (for PGE2, PGF_2α_, IL-6, and Hs-CRP). *p* value ^2^: within-Intervention group comparison of variables resulted from paired sample *t*-test (for PGE2, PGF_2α_, IL-6, and Hs-CRP). *p* value ^3^: within-Placebo group comparison of variables resulted from paired sample t-test (for PGE2, PGF_2α_, IL-6, and Hs-CRP).

## Data Availability

The datasets used and analyzed during the current study are available from the corresponding author on reasonable request.

## Supplementary Materials

The following supporting information can be downloaded at: https://www.mdpi.com/article/10.3390/nu16234026/s1, Table S1: Original data of painfulness scores; Table S2: Original data of discomfort scores.
