# Supplementary material for: Impact of Adlay-Based Formula on Pain and Discomfort in Women with Dysmenorrhea: A Randomized Controlled Trial
Source: Nutrients. 2024 Nov 24;16(23):4026. doi: 10.3390/nu16234026 (PMC11643813; doi:10.3390/nu16234026)
Supplement: Supplementary file 1 [file nutrients-16-04026-s001.zip › nutrients-3314186-supplementary.pdf]

Supplementary Table S1. Original data of painfulness scores

	Intervention				Placebo			
	Baseline		End		Baseline		End	
Items	Mean	SD	Mean	SD	Mean	SD	Mean	SD
1	2.78	0.87	2.13	0.82	2.89	0.63	2.53	0.83
2	2.47	1.16	1.90	0.96	2.43	0.88	2.26	0.90
3	1.69	1.01	1.37	0.67	1.46	0.66	1.82	1.00
4	1.53	0.81	1.17	0.46	1.54	0.85	1.65	0.95
5	2.81	1.09	2.20	0.92	2.77	1.00	2.65	0.98
6	1.42	0.91	1.23	0.57	1.43	0.65	1.44	0.82
7	1.67	0.89	1.57	0.90	1.51	0.70	1.76	0.96
8	3.19	0.75	1.98	0.84	2.83	0.75	2.32	0.88
9	3.31	0.71	2.10	0.68	2.89	0.83	2.53	0.96
10	1.58	0.69	1.23	0.43	1.40	0.65	1.39	0.90
11	1.47	0.94	1.20	0.55	1.69	1.05	1.47	0.90
12	2.75	1.16	2.00	0.98	2.63	0.91	2.38	1.13
13	2.22	1.15	1.30	0.53	1.71	0.89	1.76	1.05
14	1.44	0.88	1.23	0.43	1.40	0.81	1.44	0.86
15	1.56	0.94	1.23	0.63	1.69	0.96	1.65	1.01

Supplementary Table S2. Original data of discomfort scores

	Intervention				Placebo			
	Baseline		End		Baseline		End	
Items	Mean	SD	Mean	SD	Mean	SD	Mean	SD
1	2.14	0.93	1.63	0.76	1.83	0.71	1.52	0.68
2	2.33	0.76	1.93	0.78	2.03	0.82	1.65	0.66
3	2.36	0.76	2.00	0.83	2.03	0.62	1.77	0.72
4	3.06	0.83	2.27	0.83	2.40	0.77	2.29	0.86
5	2.19	0.92	1.73	0.83	1.89	0.87	1.65	0.75
6	2.58	0.94	2.03	0.81	2.23	0.84	1.87	0.72
7	1.97	1.18	1.90	0.96	1.77	0.84	1.74	0.77
8	1.69	0.95	1.70	0.95	1.54	0.66	1.48	0.68
9	1.86	1.02	1.17	0.38	1.54	0.85	1.52	1.00
10	2.44	0.77	1.70	0.75	2.09	0.89	1.81	0.83
11	2.75	0.81	2.50	0.86	2.31	0.83	2.26	1.03
12	1.56	0.77	1.33	0.61	1.29	0.52	1.19	0.40
13	1.39	0.55	1.27	0.52	1.23	0.55	1.35	0.66
14	2.00	0.89	1.40	0.67	1.86	0.97	1.48	0.68
15	1.17	0.45	1.17	0.38	1.23	0.65	1.29	0.53
